# Editorial Chit-Chat

**Published:** 1879-07

**Authors:** 


### Editorial Chit-Chat.

"BODINES."—Have you read it? If not, purchase a copy and so do, when you will know as much about "camping out" as we do.

DELINQUENTS.—Many of our subscribers are in arrears for the present year. Please send in your fifty cents at once. Three, two and one cent postage stamps taken for subscription. Do not send stamps of a higher denomination.

THE CAMPING BOOK.—Any of our friends at a distance, desiring to read "Bodines," and wishing to have us act as their agent in procuring the book for them, may send to us $1.50, when we will have the book sent them postage paid.

BUSHELS OF CATS.—A chap, in a recent number of the *Husbandman*, advertised for sale five hundred bushels of cats! Is not this an indiscretion on the part of the sausage makers, to permit their material to be advertised in this manner?

THE CREMATION SOCIETY now has two lady members. Three hundred dollars have been subscribed toward the building of a furnace. Any persons desiring to join the society or to contribute funds to the building of the furnace, can do so by applying to Rev. Thos. K. Beecher, or to the Editor of this journal.

BEEF-WINE AND IRON.—We wish to call the attention of medical men to the excellence of this preparation. We have used it much in our practice, and can recommend it as an excellent tonic for convalescents, or for all ailments where a nutritive stimulant is indicated. We call attention to the announcement of it by Wm. R. Warner & Co.

THE LOAN EXHIBITION.—This exhibition, introduced through the energy of the ladies of this city, in aid of the "Home for the Aged," has proved a splendid success. Not only has it resulted in a substantial benefit to the Home, but has been a real pleasure to our citizens.

Mr. F. G. Hall, the President of the Exhibition, proved himself to be a capable officer. To his indefatigable exertions is largely due the excellence of the exhibition and the promptness with which it was made ready for visitors.

THE LAWN MOWER.—Can anybody tell why such an exorbitant price is asked for lawn mowers? Is there any sense or reason in asking eighteen dollars for a machine that does not cost more than two dollars and a half to make? Now is there? We will hire a horse to gnaw our grass short before consenting to the imposition of paying such a price for a mower.

CLUB 101.—Many a day has passed since that popular club flourished. To-day, we were astonished to find that it had revived again, and that a tall and graceful conductor on the N. C. Railway was wearing the mystic figures "101" upon his coat collar. We welcome the gentleman as an exemplary member of an ancient and honorable order, and doubt not that many of the mystic tie will avail themselves of the privilege of riding with the member who so conspicuously exhibits the emblem of the order.

UNCONSCIOUS HILARITY. — "Here's Uncle Ben! O, here's Uncle Ben!" shouted a young lady as the train on the Northern Central drew up to the station at Troy. Everybody rushed to her side of the car to take a glimpse at "Uncle Ben," who proved to be a round faced, good natured looking chap, that blushed and stammered a welcome to his gushing niece. We fancied a shade of satisfaction passed over "Uncle Ben's" countenance as the train moved on and carried the crowd of laughing and curious gazers with it.

HOME PRESCRIPTIONS.—Those abominable home prescription cases, put up by dealers in homeopathic medicines, have done an incalculable amount of mischief. They are in use in a great many families, the mother usually being the prescriber for the many little ailments of her children. As the tendency for all acute diseases is to get well, the mother ascribes the recovery to the efficacy of her medicines, and so becomes emboldened to risk the chance of prescribing for more serious ailments. And right here comes in the harmful consequences of such meddlesome interference. The child becomes rapidly worse, while the mother, not recognizing the dangerous symptoms, plies the little one with the inert pellets that months before had parted with any vestige of the tinctures with which they were moistened, and the time for successfully combatting the disease at the hands of a physician is allowed to slip by.

We are impelled to condemn this indiscriminate home prescribing, from the circumstance of having witnessed two deaths recently that were directly attributable to this cause. It is a very hazardous piece of economy that impels one to trifle with the ailments of children. Better by far pay your family physician for an unnecessary call than to permit the time for a successful prescription to pass.

---

DIXEY ON ART.—Mr. Dixey was invited to take charge of the Art Rooms for a day in the Loan Exhibition. During his superintendence a countryman found his way to the gallery; looked about unconcernedly until his eyes rested upon the large classical painting of Simeon and the Child.

"What's that?" enquired the visitor. "That," said Dixey, "why that is Rip Van Winkle, with Gretchen's baby, painted by Signor Blitz!"

"O! yes!" said the countryman, as he walked away, satisfied with the information.

---

POLITENESS.—A gentleman seated immediately behind two well behaved young ladies in a railway car, was attacked with a fit of sneezing. The young women thinking doubtless that the gentleman was "taking cold" from the open window at which they were sitting, promptly closed it. This was such a remarkable instance of urbanity and politeness upon the part of the young travelers, that the gentleman thought it worth while to relate it to us, particularly as he had traveled widely and had never encountered an act like that before.

---

THE DWIGHT INQUEST.—The most beastly depredation ever committed upon a dead body, in modern times, was the one enacted by a few so-called Life Insurance Companies, upon the body of Colonel Dwight, of Binghamton, N. Y. The Colonel had insured his life for a large sum of money, in several companies. He paid the premiums, was taken sick a short while afterward, and died. His physicians, in the presence of medical examiners sent by the companies, made an autopsy, and found that his death was the result of natural causes. Upon this opinion, one reputable company (the Equitable of N. Y.) promptly paid fifty thousand dollars for which they insured the life. Several other companies—whose names we would like to publish if we had them at hand—sought to escape their obligations to the heirs by proclaiming that the Colonel had committed suicide; that he had hanged himself in fact, and then having deliberately cut himself down, hid the rope, spread himself out calmly upon the bed, called in his friends and died.

This statement seems exaggerated, but this is precisely the defense set up by said companies.

If these life insurance companies do not learn to pay their death claims as soon as presented, and cease contesting the payment of losses, the people will learn the utter uselessness of investing money in such abominable swindling concerns.

---

BE CAUTIOUS.—When you invite a party of ladies to your camp in the woods, and offer inducements in the way of singing of wood-robins, whippoorwills and other birds of melody, the screeching and hooting of owls, and the bounding of flying squirrels on the tents at night; be cautious, else may you be compelled to sit up half the night throwing potatoes on the tent roofs, and making your throat sore with all manner of unearthly screeches, to satisfy your visitors that they have not been deceived. Nothing short of the complete programme will ever satisfy them. One of the campers is suffering still from a severe cold contracted at four o'clock in the morning, while climbing an elm tree to imitate the crawling and wailing of a catamount.

We are glad to know however that some folks will remain at a show until the advertised performance is over.

---

SUNDAY NEWSPAPERS.—Generally, this sort of a paper is a nuisance, not fit to a place in a household. They pander to the wants of the lower classes of community, and abound in blood-and-thunder stories and personal inuendoes. It is refreshing, therefore, to pick up a decent Suday paper that furnishes you with desirable reading matter with no mixture of the bad. We approve of and recommend our *Sunday Telegram*.

---

WARNER'S PILLS AND PARVULES.—Our professional readers will not fail to notice Wm. R. Warner & Co's new advertisement.—We have used their pills and parvules in our own practice finding them convenient and efficacious.

